# Comparison of genetic association strategies in the presence of rare alleles

**DOI:** 10.1186/1753-6561-5-S9-S32

**Published:** 2011-11-29

**Authors:** Jestinah M Mahachie John, Tom Cattaert, Lizzy De Lobel, François Van Lishout, Alain Empain, Kristel Van Steen

**Affiliations:** 1 Systems and Modeling Unit, Montefiore Institute, University of Liege, Grande Traverse 10, 4000 Liège, Belgium; 2 Bioinformatics and Modeling, GIGA-R, University of Liege, Avenue de l’Hôpital 1, 4000 Liège, Belgium; 3 Department of Applied Mathematics and Computer Science, Ghent University, Krijgslaan 281 S9, 9000 Ghent, Belgium

## Abstract

In the quest for the missing heritability of most complex diseases, rare variants have received increased attention. Advances in large-scale sequencing have led to a shift from the common disease/common variant hypothesis to the common disease/rare variant hypothesis or have at least reopened the debate about the relevance and importance of rare variants for gene discoveries. The investigation of modeling and testing approaches to identify significant disease/rare variant associations is in full motion. New methods to better deal with parameter estimation instabilities, convergence problems, or multiple testing corrections in the presence of rare variants or effect modifiers of rare variants are in their infancy. Using a recently developed semiparametric strategy to detect causal variants, we investigate the performance of the model-based multifactor dimensionality reduction (MB-MDR) technique in terms of power and family-wise error rate (FWER) control in the presence of rare variants, using population-based and family-based data (FAM-MDR). We compare family-based results obtained from MB-MDR analyses to screening findings from a quantitative trait Pedigree-based association test (PBAT). Population-based data were further examined using penalized regression models. We restrict attention to all available single-nucleotide polymorphisms on chromosome 4 and consider Q1 as the outcome of interest. The considered family-based methods identified marker C4S4935 in the *VEGFC* gene with estimated power not exceeding 0.35 (FAM-MDR), when FWER was kept under control. The considered population-based methods gave rise to highly inflated FWERs (up to 90% for PBAT screening).

## Background

Analyzing the effects of genes and/or environmental factors on the development of complex diseases is a great challenge from both the statistical and the computational perspectives. Calle et al. [1,2] recently developed the model-based multifactor dimensionality reduction (MB-MDR) technique, which tackles association and interaction analysis by assigning genotype cells to different risk categories. This method is also applicable to one-dimensional screening. Currently, the MB-MDR approach uses permutation testing to assess significance [3], thereby also correcting for multiple testing. An additional major problem arises when associations between a trait of interest and rare variants are targeted. In this context, it is unclear which of the family-based or population-based designs will be more advantageous. Also, traditional regression methods break down because parametric assumptions are hardly fulfilled for rare variants [4]. In this paper, we explore the utility of several methods, both parametric and nonparametric, to test for or model genetic associations using population-based and family-based data from Genetic Analysis Workshop 17 (GAW17).

## Methods

### Data set and quantitative trait association analysis

The data provided by GAW17 include a subset of genes grouped according to pathways that had sequence data available in the 1000 Genomes Project. Effect sizes for coding variants within these genes were assigned using PolyPhen and SIFT predictions of the likelihood that the variant would be deleterious. Two hundred replicates were generated. Our analyses involve the quantitative trait Q1, which was simulated as a normally distributed phenotype. Furthermore, we restrict attention to the available single-nucleotide polymorphisms (SNPs) on chromosome 4 (944 in total). All simulated singular SNP effects (SNPs C4S1861, C4S1873, C4S1874, C4S1877, C4S1878, C4S1879, C4S1884, C4S1887, C4S1889, and C4S1890 in the *KDR* gene and C4S4935 in the *VEGFC* gene) are assumed to be additive on the quantitative trait scale, such that each copy of the minor allele increases or decreases the mean trait value by an equal amount. In addition, values of Q1 were simulated to be higher in smokers, and the listed variants in the *KDR* gene were involved in *KDR*-smoking interaction effects on the trait. There are 944 markers in 81 genes on chromosome 4. The sample size for both population- and family-based data is 697 with family data comprising 8 families with 202 founders and 3 offspring generations. The founders were randomly sampled from the unrelated individuals data set, and genotypes of offspring were sampled using Mendelian inheritance. It should be noted that genetic information is the same for all replicates; only phenotype and smoking status differ.

For the family data, we compare the performance of the MB-MDR approach (family-based, FAM-MDR) to the association test (PBAT) screening [5-7] (version 3.61), whereas for unrelated individuals we compare the MB-MDR approach to penalized regression (the penalized package in R, v. 2.9). Power is estimated on the basis of rejection of the null hypothesis for the SNP under investigation, whereas the family-wise error rate (FWER) is estimated on the basis of rejection of the null hypothesis for any of the SNPs with no effect. In addition, we reevaluated power and FWER by collapsing rare variants in the genes of chromosome 4.

In the following subsections, we briefly describe the main characteristics of the approaches we consider in this comparative study.

### MB-MDR modeling

The MB-MDR technique for one dimension involves three steps. First, each marker’s genotype cells are assigned to one of three categories—high risk (H), low risk (L), or no evidence (O)—on the basis of the result of association tests (*t* tests) on each of the individual cells versus all other cells with the response variable, using a liberal *p*-value threshold of 0.1 [3]. If this threshold is not attained for whatever reason, the cell is labeled O. Next, an association test is performed with the new predictor variable *X* in {H, L, O} on the outcome variable. Association with the trait is investigated by testing H versus L [3] using a *t* test. In the last step, permutation-based step-down max *T* adjusted *p*-values [8] with 999 replicates are computed to assess significance over all considered marker sets, theoretically ensuring control of FWER at 5%. We also implement the step-down min *P* procedure [8], based on 999,999 replicates.

The MB-MDR approach has been adapted to accommodate family-based study designs and uses principles of genome-wide rapid association using mixed model and regression [9]. In particular, the MB-MDR approach for families (FAM-MDR [10]) first involves performing a polygenic analysis using the complete pedigree structure. Then MB-MDR (for unrelated individuals) is applied to familial correlation-free residuals obtained from the polygenic modeling.

### Family-based association testing for family-based designs

The PBAT screening approach of Van Steen et al. [7] is adopted to identify the top 10 most powerful genotype-phenotype combinations and to independently test these using the family-based association test (FBAT) statistic [6]. To be more in line with our MB-MDR analyses, we report results of the dominant genetic model rather than the additive genetic model. Family 7 was split into nuclear families for better handling by PBAT. Type I errors are Bonferroni controlled.

### Penalized regression for population-based designs

To select the 10 most interesting predictors for Q1, we also apply a least absolute shrinkage and selection operator (LASSO) penalized regression [11]. We decrease the penalizing parameter *λ* with a precision of 0.001 to obtain at least 10 (nonzero) markers in the model, to be in line with PBAT screening. However, sometimes a few more markers were selected (maximum of 12). The covariates Sex, Age, and Smoke are fixed and unpenalized in the regression model. We repeated this analysis for each replicate to obtain a screening technique for the main effects. After this screening procedure, the selected markers were put in a linear regression model to test for association with Q1, again fixing Age, Sex, and Smoke in the model. *P*-values are Bonferroni corrected for the number of markers in the data.

### Gene-based collapsing method

Following Li and Leal [12] for discrete traits and Morris and Zeggini [13] for quantitative traits, we collapsed variants with a minor allele frequency (MAF) less than 0.01 within each gene into a single variable coded 0 for absence and 1 for presence of at least one variant allele in an individual. The reported MAFs were evaluated using all individuals separately within each considered study design.

## Results

Additional file 1 presents estimated power levels for association of important main effects with Q1 using the aforementioned methods. We observe that the MB-MDR approach for unrelated individuals has some power (0.14 for max *T* and 0.34 for min *P*) to find C4S1878, the marker with the largest MAF (0.16), but also elevated FWER estimates (0.13 and 0.50, respectively). With penalized regression, the highest power is achieved for markers C4S1884 (MAF = 0.02) and C4S1877 (MAF < 0.001), irrespective of whether a gene-collapsing method was adopted or not. However, these results are downplayed by the inflation of the corresponding FWER (>0.3). PBAT exhibits extremely high power (0.94) to detect C4S4935 (MAF < 0.001) but also has an extreme FWER of 0.895. When a correction is made for the presence of linkage, interestingly, PBAT’s power drops to 0 and its FWER drops to 0.015. On the other hand, the FAM-MDR approach has only limited power (0.18 for max *T* and 0.17 for min *P*) to detect C4S4935 but keeps the FWER under control.

**Figure 1:**
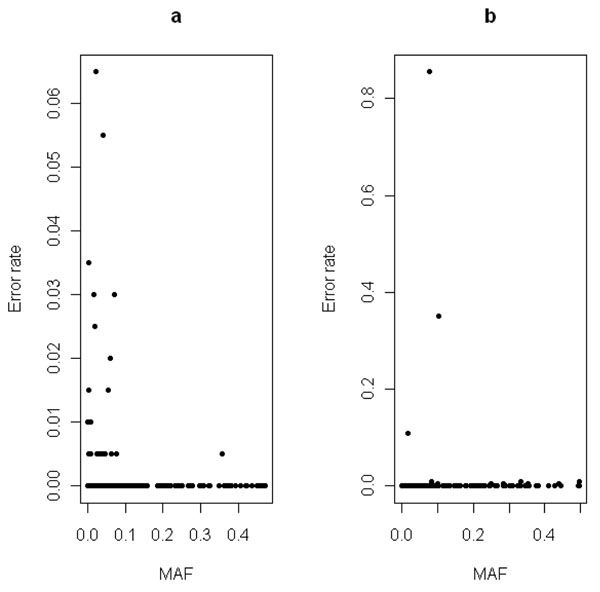
**Marker-specific error rates**. Marker-specific error rates as a function of minor allele frequency (MAF) for all nonfunctional markers. (a) MB-MDR using max *T* on unrelated individuals and (b) PBAT with default options.

A graphical representation of the relation between error rates for nonfunctional markers and the corresponding markers’ MAFs is given in Figure 1.

Finally, collapsing rare variants increases the estimated power of the MB-MDR approach on unrelated individuals, both for the common variants C4S1878 (0.375 for max *T* and 0.38 for min *P*) and C4S1884 (0.205 for max *T* and 0.155 for min *P*) on the *KDR* gene and for the collapsed variable obtained from the rare variants on the *KDR* gene (0.355 for max *T* and 0.47 for min *P*). For the FAM-MDR analysis for families, collapsing increases the power to detect the variant C4S4935 on the *VEGFC* gene (0.275 for max *T* and 0.345 for min *P*). FWER for unrelated individuals remains high, whereas for family data FWER is under control.

## Discussion

Using the considered methods, we observed that different markers were highlighted in unrelated individuals versus families. Given the extent of monomorphic and nearly monomorphic causal variants with Q1 on chromosome 4, it is not surprising that none of the adopted methods perform satisfactorily in identifying genetic effects in the presence of rare variants. In particular, marker C4S4935 (Additional file 1) has only one heterozygous individual in the unrelated individuals data, and hence no method will be powerful enough to highlight this marker. However, this heterozygous individual was selected as a founder and propagated in one of the eight families, leading to an increased number of copies of the variant allele and consequently increased power to identify C4S4935 in the family data.

As a side remark, we also investigated whether the MB-MDR approach was able to identify the gene-smoking interaction effect present in the data. It is not surprising that detecting it was virtually powerless. Six out of 10 SNPs showing gene-environment interaction with smoking have such extremely low MAFs that no homozygous individuals for the rare allele and only one heterozygous individual were observed. Hence, for these SNPs, information about their potential to change the effects of smoking on Q1 is basically nonexistent because the one heterozygous individual is either a smoker or a nonsmoker. For unrelated individuals, none of the 944 markers are monomorphic, whereas 403 of the markers are monomorphic in the family data, leaving only 541 markers of interest in 77 genes. This can be explained by existing founder effects.

The beauty of the permutation-based corrective method for multiple testing used in the MB-MDR approach is that it tackles the issue of testing a large number of marker sets for evidence of gene-gene interactions with the trait, by controlling FWER at 5% [3]. We argue that the uncontrolled FWER levels might be a direct consequence of the distributional properties of association test statistics involving rare variants and their effect on the validity of both the adopted testing procedure and the applied multiple testing corrective methods. For instance, max *T* and min *P* adjusted *p*-values are known to be similar when the test statistics are identically distributed. When this is not the case, max *T* adjustments may be unbalanced such that not all tests equally contribute to the adjustment, leading to suboptimal power. The drawback of the min *P* implementation is that it is less computationally tractable than the max *T* approach and that a large number of permutations are needed to detect possible improved effects over max *T* implementations. A promising alternative approach may be the max *T* scaled method of Nacu et al. [14]. This method adjusts each test statistic by subtracting its null mean and dividing by its null standard deviation, leading to comparable null distributions. The max *T* scaled method can be considered a parametric and fast version of the min *P* method and requires a comparable number of permutations as the max *T* approach.

The total contribution to FWER of markers in linkage disequilibrium with functional markers (*r*^2^ > 0.9) is only 0.01; hence linkage disequilibrium can be ruled out as an explanation of the increased FWER. In contrast, rare effects seem to be the major cause of the observed elevated FWER estimates. This is further supported by the observation that 1 out of 200 simulated replicates gives an erroneous result among the markers with MAF > 0.1 (Figure 1a). Under the assumption that the markers with MAF < 0.1 (90% of the data) and the markers with MAF > 0.1 (10% of the data) behave similarly, we expect that 1 + 9 = 10 out of 200 replicates will give rise to an erroneous result (all markers considered). Hence the FWER would indeed be controlled at 5%. The same reasoning can be adopted to explain the conservativeness of the PBAT approach in the presence of rare variants, especially when the empirical variance option (test of no association in the presence of linkage) is used. In effect, the apparent liberal results observed in Pedigree based association testing with default screening parameters seem to be caused by a limited number of problematic markers (Figure 1b). Omitting marker C4S4694 (with MAF = 0.08) from the analysis indeed decreases the FWER from 0.895 to 0.445. When we remove three additional markers with moderate MAFs showing errors in multiple replicates, FWER tends to 0.04.

Joint application of the MB-MDR approach and gene collapsing leads to increased power, which can be explained by both the reduced multiple testing burden (note the increase in power for the common variants) and the creation of variables that exhibit larger amounts of information. For unrelated individuals, of the 944 markers, 199 have MAF ≥ 0.01 and 745 have MAF < 0.01, and they are collapsed into 72 gene-specific variables. For the families, of the 541 nonmonomorphic markers, 227 have MAF ≥ 0.01 and 314 have MAF < 0.01, and they are collapsed into 60 gene-specific variables. Surprisingly, FWER increased when the MB-MDR approach was applied to unrelated individuals. Notably, one of the drawbacks of adopting collapsing methods is that singular effects for rare variants cannot be distinguished from global gene effects.

## Conclusions

We compared several genetic association strategies to detect main effects, including the MB-MDR approach, PBAT screening, and penalized regression. Although none of the methods exhibited sufficient power to detect rare variants, remarkable differences were observed between these methods within and between study designs. At this point it is not clear whether these differences are due to the particular way the genetic effects were simulated in the family-based or population-based data or whether they are actually due to the methods themselves. However, most important, we postulate that the rarity of certain marker alleles hampers the validity of model assumptions and distributional properties of test statistics as well as assumptions underlying some commonly used measures to correct for multiple testing or to control false-positive rates.

## Competing interests

The authors declare that there are no competing interests.

## Authors’ contributions

JMMJ, TC and KVS participated in the statistical analyses related to the MB-MDR technique. LDL and KVS participated in the analyses related to PBAT and penalized regression. FVL participated in optimizing the MB-MDR software for this project. AE participated in data handling and manipulation. All authors read and approved the final manuscript.

## Supplementary Material

Additional file 1 **Table 1 - Power to detect functional markers and FWER** Power and FWER results are shown for the MB-MDR approach and penalized regression on unrelated individuals, and FAM-MDR and FBAT results are shown for family data, both on original and collapsed chromosome 4 data. Power values greater than 0.1 are indicated in bold, and FWER values greater than 0.1 are indicated in italic. ^a^ At least 10 markers are selected using penalized regression, with Sex, Age, and Smoke as the fixed covariates. ^b^*P*-values are Bonferroni-corrected according to the total number of markers. Sex, Age, and Smoke are fixed covariates in the final model. ^c^ PBAT screening uses FBAT statistic to test the null hypothesis of no association in the presence of linkage. Click here for file
