# Supplementary material for: Comparison of genetic association strategies in the presence of rare alleles
Source: BMC Proc. 2011 Nov 29;5(Suppl 9):S32. doi: 10.1186/1753-6561-5-S9-S32 (PMC3287868; doi:10.1186/1753-6561-5-S9-S32)
Supplement: Additional file 1 — Table 1 - Power to detect functional markers and FWER Power and FWER results are shown for the MB-MDR approach and penalized regression on unrelated individuals, and FAM-MDR and FBAT results are shown for family data, both on original and collapsed chromosome 4 data. Power values greater than 0.1 are indicated in bold, and FWER values greater than 0.1 are indicated in italic. a At least 10 markers are selected using penalized regression, with Sex, Age, and Smoke as the fixed covariates. bP-values are Bonferroni-corrected according to the total number of markers. Sex, Age, and Smoke are fixed covariates in the final model. c PBAT screening uses FBAT statistic to test the null hypothesis of no association in the presence of linkage. [file 1753-6561-5-S9-S32-S1.doc]

# Comparison of genetic association strategies in the presence of rare alleles

Jestinah M Mahachie John^1,2^*, Tom Cattaert^1,2^*, Lizzy De Lobel^3^, François Van Lishout^1,2^, Alain Empain^2^, Kristel Van Steen^1,2§^

^1^Systems and Modeling Unit, Montefiore Institute, University of Liege, Grande Traverse 10, 4000 Liège, Belgium

^2^Bioinformatics and Modeling, GIGA-R, University of Liege, Avenue de l'Hôpital 1, 4000 Liège, Belgium

^3^Department of Applied Mathematics and Computer Science, Ghent University, Krijgslaan 281 S9, 9000 Gent, Belgium

*These authors contributed equally to this work

^§^Corresponding author

Email addresses:

JMMJ: [jmahachie@ulg.ac.be](mailto:jmahachie@ulg.ac.be)

TC: [tom.cattaert@ulg.ac.be](mailto:tom.cattaert@ulg.ac.be)

LDL: [lizzy.delobel@ugent.be](mailto:lizzy.delobel@ugent.be)

FVL: [f.vanlishout@ulg.ac.be](mailto:f.vanlishout@ulg.ac.be)

AE: [alain.empain@ulg.ac.be](mailto:alain.empain@ulg.ac.be)

KVS: [kristel.vansteen@ulg.ac.be](mailto:kristel.vansteen@ulg.ac.be)

## Table 1 - Power to detect functional markers and FWER

Power and FWER results are shown for MB-MDR and Penalized Regression on unrelateds, and FAM-MDR and FBAT on family data, both on original and collapsed chromosome 4 data Power values >0.1 are indicated in bold, and FWER values >0.1 are indicated in italic.

| MB-MDR | marker | C4S1878 | C4S1884 | C4S1861 | C4S1873 | C4S1874 | C4S1877 | C4S1879 | C4S1887 | C4S1889 | C4S1890 | C4S4935 | FWER |
| --- | --- | --- | --- | --- | --- | --- | --- | --- | --- | --- | --- | --- | --- |
|  | MAF | 0.16499 | 0.02080 | 0.00215 | 0.00072 | 0.00072 | 0.00072 | 0.00072 | 0.00072 | 0.00072 | 0.00215 | 0.00072 |  |
|  | maxT | **0.14** | 0.03 | 0 | 0.005 | 0.005 | 0.015 | 0 | 0 | 0.015 | 0 | 0 | *0.13* |
|  | minP | **0.335** | 0.06 | 0 | 0 | 0 | 0.015 | 0 | 0 | 0.015 | 0 | 0 | *0.495* |
| MB-MDR  collapsed | marker | C4S1878 | C4S1884 | KDR collapsed | | | | | | | | VEGFC collapsed | FWER |
|  | maxT | **0.375** | **0.205** | **0.355** | | | | | | | | 0.01 | *0.69* |
|  | minP | **0.38** | **0.155** | **0.47** | | | | | | | | 0.005 | *0.735* |
| Penalized  Regression | marker | C4S1878 | C4S1884 | C4S1861 | C4S1873 | C4S1874 | C4S1877 | C4S1879 | C4S1887 | C4S1889 | C4S1890 | C4S4935 | FWER |
|  | Screen^2^ | 0.120 | 0.515 | 0.020 | 0 | 0.02 | 0.87 | 0 | 0.015 | 0.87 | 0.005 | 0.215 |  |
|  | Power^3^ | 0.03 | **0.185** | 0 | 0 | 0 | **0.57** | 0 | 0 | 0 | 0 | 0 | *0.32* |
| Penalized  Regression  Collapsed | Marker | C4S1878 | C4S1884 | KDR collapsed | | | | | | | | VEGFC collapsed | FWER |
|  | Screen^2^ | 0.195 | 0.565 | 0.755 | | | | | | | | 0.305 |  |
|  | Power^3^ | 0.06 | **0.11** | **0.35** | | | | | | | | 0.025 | *0.48* |
| FAM-MDR | marker | C4S1878 | C4S1884 | C4S1861 | C4S1873 | C4S1874 | C4S1877 | C4S1879 | C4S1887 | C4S1889 | C4S1890 | C4S4935 | FWER |
|  | MAF | 0.13702 | 0.01937 | 0.00215 | 0.00143 | 0 | 0 | 0 | 0 | 0 | 0.00072 | 0.02224 |  |
|  | maxT | 0 | 0 | 0 | 0.005 | 0 | 0 | 0 | 0 | 0 | 0 | **0.18** | 0.065 |
|  | minP | 0 | 0 | 0 | 0.005 | 0 | 0 | 0 | 0 | 0 | 0 | **0.165** | 0.04 |
| FAM-MDR  collapsed | marker | C4S1878 | C4S1884 | KDR collapsed | | | | | | | | C4S4935 | FWER |
|  | maxT | 0 | 0 | 0 | | | | | | | | **0.275** | 0.06 |
|  | minP | 0 | 0 | 0 | | | | | | | | **0.345** | 0.06 |
| PBAT | marker | C4S1878 | C4S1884 | C4S1861 | C4S1873 | C4S1874 | C4S1877 | C4S1879 | C4S1887 | C4S1889 | C4S1890 | C4S4935 | FWER |
|  | Default | 0 | 0 | 0 | 0 | 0 | 0 | 0 | 0 | 0 | 0 | 0.94 | 0.895 |
|  | Linkage^1^ | 0 | 0 | 0 | 0 | 0 | 0 | 0 | 0 | 0 | 0 | 0 | 0.015 |

^1^ PBAT screening uses FBAT test statistic to test the null hypothesis of no association in the presence of linkage

^2^ At least 10 markers are selected using penalized regression. with sex. age and smoke as fixed covariates.

^3^ P-values are Bonferroni corrected according to the total number of markers. Sex. age and smoke are fixed covariates in the final model.
